# Health-Related Quality of Life of Hypertension Patients: A Population-Based Cross-Sectional Study in Chongqing, China

**DOI:** 10.3390/ijerph16132348

**Published:** 2019-07-03

**Authors:** Meng Xiao, Fan Zhang, Nanzi Xiao, Xiaoqing Bu, Xiaojun Tang, Qian Long

**Affiliations:** 1 School of Public Health and Management, Research Center for Medicine and Social Development, Collaborative Innovation Center of Social Risks Governance in Health, Chongqing Medical University, Chongqing 400016, China; 2 Global Health Research Center, Duke Kunshan University, Kunshan 215316, China

**Keywords:** hypertension, health-related quality of life, SF-36, cross-sectional study

## Abstract

*Purpose*: Hypertension is a major risk factor for cardiovascular diseases and stroke, and it requires lifelong medication. This study aimed to investigate the factors impacting on Health-Related Quality of Life (HRQoL) among hypertensive patients in Chongqing, China, and to provide evidence-based strategies to improve their HRQoL. *Methods*: This cross-sectional survey was conducted in Chongqing, China. Of 600 randomly selected patients, 586 patients agreed to participate and 567 patients completed the survey. A SF-36 (Medical Outcomes Study (MOS) Short Form Health Survey questionnaire) that included eight domains: physical functioning, role limitations due to physical problems, body pain, general health, vitality, social function, role limitations due to emotional problems, and mental health was used to measure HRQoL. Linear regressions were used; each domain of HRQoL was measured in the stratification of sex. *Results*: Self-perceived relatively low economic burden caused by hypertension and regular physical activity had a positive impact on HRQoL (*p* < 0.05) for both men and women. For women, younger age was associated with higher scores of measuring physical functioning and body pain. Living with more than three family members had a positive impact on domains, including physical functioning. Emotional self-regulation had a positive association with women’s mental health. Alcohol use for men was associated with higher scores in physical and mental health measures, and emotional self-regulation showed some positive impact on general health. *Conclusion*: Perceived economic burden caused by hypertension was the most common factor impacting on patients’ HRQoL. Female patients were more susceptible when compared to male patients. Health intervention strategies need to be further explored and adapted to the context of improving HRQoL for patients who suffer from hypertension and other chronic non-communicable diseases.

## 1. Introduction

Cardiovascular disease, including hypertension costs, accounted for about 10% of the total healthcare spending in certain low- and middle-income countries, according to the estimation of global report on non-communicable diseases in 2013 [1]. In China, the prevalence of hypertension increased from 15.6% in 1991 to 20.9% in 2011 [2]. In addition, hypertension is a major risk factor for cardiovascular diseases [3] and stroke [4], and it requires lifelong medication [5], which is associated with heavy socio-economic burdens on individuals and health systems.

Health-related quality of life (HRQoL) is an important measure for non-communicable chronic diseases (NCDs), in terms of physical and mental health outcomes [6,7,8]. There are multiple assessment tools for measuring health-related quality of life, for example, SF-36 (Medical Outcomes Study Short Form Health Survey questionnaire), EQ-5D (Euro Qol), and WHOQOL (The World Health Organization Quality of Life). SF-36 is a self-administered questionnaire and it has been widely used in the field of HRQoL study [9]. The essential components of health related life quality assessments include: physical health, mental health, social function, disease status, and overall health perception [10].

Chongqing is one of four municipalities that are located in southwest China with over 30,000,000 urban and rural population [11]. The prevalence rate of hypertension in Chongqing increased over the past ten years, and the estimated prevalence of hypertension was 23.9% in 2013 [12]. In Chongqing, the guideline for prevention and treatment of hypertension recommends to establish health records of hypertension patients and to have no less than four follow-up visits annually, which is also one of the components of essential public health services funded by the government [13]. 

A previous study reported that hypertension was associated with low HRQoL [14], which is a risk factor for serious health events or adverse health outcomes [15]. This study aimed to investigate factors impacting on HRQoL among hypertensive patients in Chongqing, in order to provide evidence-based strategies for improving HRQoL and health outcomes of hypertension patients. 

## 2. Methods

### 2.1. Study Sites and Sampling

**Figure 1:**
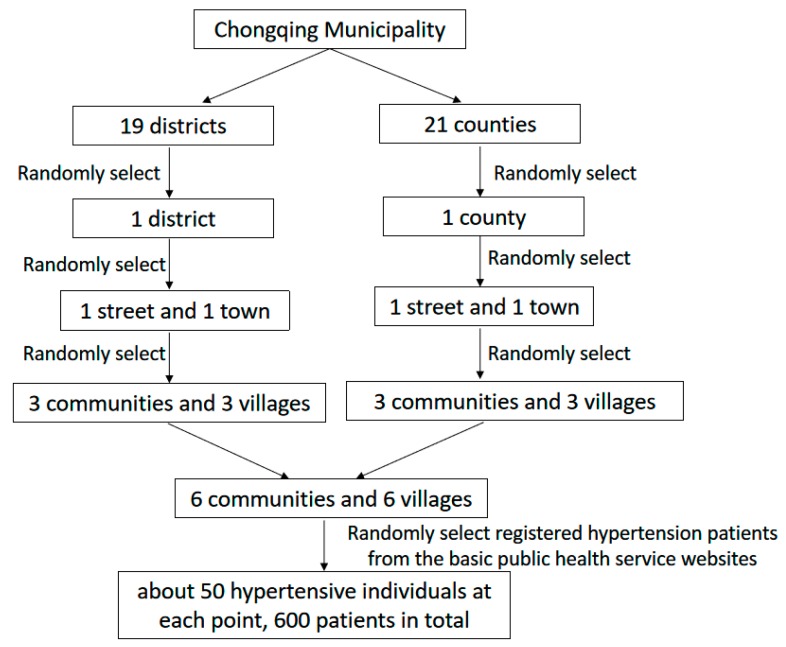
Stratified sampling.

The Chongqing municipality consists of 19 districts and 21 counties. The cross-sectional survey was conducted in Dianjiang County and the Yubei district of Chongqing, which represents relatively less developed and developed regions according to socio-economic development levels [16]. The multi-stage stratified random sampling method was used. One street and one town were randomly selected in the selected district and county. Subsequently, three communities and three villages were selected in each street and town. Finally, a total of six communities and six villages were included. The local primary health facilities are responsible for establishing hypertension management profiles for all diagnosed hypertensive patients. The hypertension management profiles include patients’ personal information, records of health checkup, and information regarding each follow-up visit. The eligible participants in this study were hypertensive patients who have been primarily diagnosed with hypertension for at least one year, were older than 18 years old, and did not have psychiatric and consciousness disorders. Hypertension patients with other chronic diseases (such as stroke, dysfunction of liver, etc.) and pregnant women were excluded. We used the hypertension management files to randomly select 50 participants from each site and downloaded their profiles after obtaining permission from chronic disease department of the primary health facilities in the selected communities, (Figure 1).

### 2.2. Data Collection

Trained postgraduate medical students and teachers conducted a face-to-face interview with patients who meet the criteria by using a structured questionnaire in addition to patients’ management files. The questions included patients’ sociodemographic information and their health-related behaviors. The village doctor or staff of the community health center invited the eligible patients coming to the community health center for the survey. A total of 586 patients participated in the survey.

SF-36 was used as a measure of HRQoL in this study. SF-36 is a self-administered scale that is widely used in the area of HRQoL study [9]. It includes 36 items and the Chinese version has been available since 1991. SF-36 contains eight domains: physical functioning (PF, 10 items were used to measure whether health conditions impede normal physiological activity), role limitations due to physical problems (RP, four items were used to measure functional limitations due to physical health problems), body pain (BP, two items were used to measure the extent of pain and the impact of pain on daily activities), general health (GH, five items were used to measure individuals’ assessment of their own health and their development trends), vitality (VT, four items were used to measure the individual’s subjective feelings about one’s own energy and fatigue), social function (SF, two items were used to measure the effects of physical and psychological problems on the quantity and quality of social activities, and evaluating the effects of health on social activities), role limitations due to emotional problems (RE, three items were used to measure functional limitations due to emotional problems), and mental health (MH, five items were used to measure four types of mental health problems, including motivation, depression, behavioral, or emotional out of control, psychological subjective feelings). The items (excluding Reported Health Transition, HT) were coded and transformed into a scale from zero (worst quality of life) to 100 (best quality of life). In addition, the eight domains were grouped into two summary components: the physical component summary (PCS, including physical function, role physical, bodily pain and general health), and the mental component summary (MCS, including vitality, social function, role emotional, and mental health).

### 2.3. Data Analysis

The explanatory variables in our study included sex, age, location (urban or rural), hypertension lasting for years, treatment methods, marital status, education level, employment status, type of medical insurance (Urban Employee Basic Medical Insurance or Urban and Rural Residence Basic Medical Insurance [17], number of family members living together, income category, self-perceived economic burden, and the self-reported physical activity, smoking, drinking, and emotional self-regulation. Age was grouped as per the World Health Organization (WHO) age classification criteria (≤59, 60–74, ≥75). Patients that were aged at 60–74 were regarded as “the young old”. The treatment methods were asked whether patients took diet control, exercise, medical treatment, and/or Chinese medicine for hypertension management and treatment. It was grouped into single treatment if patients reported one of those methods; it was grouped into multiple treatment if patients reported two or more methods. Income category was divided into three equal parts according to annual per capital income: low, medium and high.

The data were double entered into EpiData 3.1 software (EpiData Associations, Odense, Denmark) and analyzed while using the STATISTICAL ANALYSIS SYSTEM (SAS) 9.1 software program (SAS Institute, Cary, NC, USA). A multi-variate regression analysis was performed to study the association between the explanatory variables and each domain for the HRQoL measures. The multivariate stepwise regression analysis was conducted to examine the factors related to HRQoL in the stratification of sex. The level of significance was set at a two-sided *p* < 0.05.

The Ethical Committee of Chongqing Medical University approved this study (approval number: 2017004). The oral consent of all participants were obtained prior to the survey.

## 3. Results

### 3.1. Demographic and Socioeconomic Characteristics

Nineteen patients out of 586 patients who agreed to participate in the survey did not complete the questionnaire. A total of 567 participants were included in the analysis. Table 1 presents patients’ demographic and socio-economic characteristics. There were more women (60.14%) than men (39.86%) in this study. Over three-quarters of the patients (83.42%) were older than 60, and most of them lived in rural areas. Most of them (57.32%) had primary education and around one-third of patients (36.33%) were illiteracy. Half of them (55.38%) were employed. Around two-thirds of the patients (63.49%) had a diagnosis of hypertension for over five years. More than half of the patients (53.97%) used at least two methods to control their blood pressure. A vast majority of patients (78.13%) were married and more than half (58.73%) lived with two family members or less (including themselves). More than half of patients (57.67%) perceived heavy economic burden that is caused by hypertension. The great majority of the patients did physical activity at least once a week lasting for over 30 minutes, did not smoke or drink alcohol, and most of them had emotional self-regulation, according to their report.

### 3.2. SF-36 Measurement

Table 2 presents the explanatory variables that were statistically significantly associated with SF-36 scores by each domain and component summary (*p* < 0.05). Self-perceived economic burden that is caused by hypertension showed the association with all the domains. Sex was associated with Physical Functioning (PF), Body Pain (BP), Vitality (VT), Social Function (SF), Mental Health (MH), and Physical Component Summary (PCS). Men had significantly higher scores than women for all of these six domains. Age was statistically significantly associated with PF, BP, and PCS. Younger patients had higher scores than seniors in these three domains. Physical activity played an important role in PF, Role limitations due to Physical problems (RP), and PCS. The individuals who exercised at least once a week had higher scores in these domains related to physical component. In addition, individuals who reported drinking alcohol were associated with higher scores in domains of PF, RP, BP, General Health (GH), SF, Role limitations due to Emotional problems (RE), PCS, and Mental Component Summary (MCS). Those regulated emotions actively got higher scores in the domains of GH and MH.

### 3.3. SF-36 Measurement Stratified by Sex

Table 3 presents the factors that are associated with SF-36 of eight domains and two component summaries by man and woman.

#### 3.3.1. PF (Physical Functioning)

For men, those perceived low economic burden caused by hypertension and having physical activity at least once a week were associated with higher self-assessments of scores. Women who were from a young age group, lived with more than three family members, perceived low economic burden that is caused by hypertension, and had more physical activity were associated with higher scores in PF.

#### 3.3.2. RP (Role Limitations Due to Physical Problems)

For men, those perceived low economic burden caused by hypertension, having UEBMI coverage, having physical activity at least once a week, and drinking alcohol had higher scores than their counterparts. Women who lived in urban areas, received high school or above education, lived with more than three family members, and perceived low economic burden that is caused by hypertension obtained higher scores in RP.

#### 3.3.3. BP (Body Pain)

Men who perceived low economic burden caused by hypertension, had physical activity at least once a week, and lived in urban areas got higher scores in this domain. For women, younger individuals, perceived low economic burden that is caused by hypertension, and having UEBMI coverage got higher BP scores.

#### 3.3.4. GH (General Health)

For men, those perceived low economic burden that is caused by hypertension, having UEBMI coverage, drinking alcohol, and emotional self-regulation obtained higher GH scores. For women, those perceived low economic burden caused by hypertension and well educated, got higher scores in this domain.

#### 3.3.5. VT (Vitality)

For men, those perceived low economic burden caused by hypertension, having UEBMI, and from high income group got higher VT scores. For women, those perceived low economic burden caused by hypertension and exercising at least once a week obtained higher scores.

#### 3.3.6. SF (Social Function)

Both men and women who perceived low economic burden caused by hypertension got higher SF scores. In addition, male patients who had physical activity and alcohol got higher scores in this domain.

#### 3.3.7. RE (Role Limitations Due to Emotional Problems)

Male patients who perceived relatively low economic burden caused by hypertension, had physical activity more than once a week, and drinking alcohol got higher scores. For women, those perceived relatively low economic burden caused by hypertension, living with more than three family members and living in urban areas got higher scores.

#### 3.3.8. MH (Mental Health)

For men, individuals who perceived relatively low economic burden caused by hypertension and were from high income group got higher scores. Female patients perceived relatively low economic burden caused by hypertension, using more than two treatment methods, and emotional self-regulation got higher scores in this domain.

#### 3.3.9. PCS (the Physical Component Summary)

In the physical component summary, men perceived relatively low economic burden caused by hypertension, having physical activity more than once a week, drinking alcohol, and from high income group got higher scores. Among women, those from young age group, perceived relatively low economic burden caused by hypertension, having UEBMI coverage, and living with more than three family members obtained higher PCS scores.

#### 3.3.10. MCS (the Mental Component Summary)

Male patients who perceived relatively low economic burden caused by hypertension, had alcohol, and were from high income group were related to higher scores in the mental component summary. Women perceived relatively low economic burden caused by hypertension and living in urban areas got higher scores.

## 4. Discussion

In this study, we found that hypertensive patients’ age, sex, perceived economic burden caused by hypertension, physical activity, alcohol use, and emotional self-regulation were associated with HRQoL. In the stratification of sex, self-perceived economic burden that is caused by hypertension and physical activity were the common factors impacting both male and female patients’ HRQoL.

We found that elder hypertensive patients and female patients were more likely to have poor health related quality of life, which are consistent with findings in Alexandria, Egypt [18] and Shanxi, China [19]. Some previous studies in South Korea [20] and Pakistan [21] reported that hypertensive patients from the low income group were associated with worse HRQoL. In our study, we that found low income was associated with lower scores of vitality and mental health for male patients. We asked patients’ self-perceived economic burden that is caused by hypertension in this study and found that both male and female patients’ perceived low economic burden caused by hypertension were associated with better health related quality of life. In China, NCDs patients, including hypertensive patients, require long-term treatments and many of them suffered from heavy financial burden in both urban and rural areas [22,23,24]. In 2009, the government of China launched a new round national health care reform and set the goal of universal health coverage. In 2010, three basic medical insurances, NCMS, URBMI, and UEBMI, in China have achieved near-universal coverage [25]. Previous studies in China found that basic medical insurances, particularly NCMS and URBMI, provided relatively high reimbursement proportion for inpatient care, but limited coverage for outpatient care [26,27,28].

The promotion of a healthy life style will improve hypertensive patients’ health related quality of life. Consistent with other studies, we found that regular physical exercise [25,29] had positive roles in relation to patients’ physical and social functions. In addition, we also found that regulating emotions actively had a positive association on HRQoL, although few previous studies have reported this impact on hypertensive patients. However, it is unexpected that alcohol use among male patients was associated with higher scores in physical and mental health measures, which may be attributed to local culture, given alcohol use for relaxation and other social activities [30]. Additionally, a previous study that was conducted in Hong Kong [31] even suggested that, occasional and moderate alcohol use were associated with lower mortality when compared to never drinkers.

Living with family members was also positively associated with HRQoL, particularly for female patients. Similar results were also found in previous studies. One study that was conducted in 2009 in Chongqing found that the relationship with family or friends could influence health related quality of life among hypertensive patients [32]. It was also proven that lower social support was associated with poorer health related quality of life among individuals with chronic conditions, such as hypertension among Korean American patients [33]. Family support played an important role in hypertension treatment compliance [34], and patients may derive HRQoL benefits from the emotional support [35].

This study examined the association between hypertensive patients’ demographic and socio-economic characteristics and life style and health related quality of life. However, some variables, such as smoke and alcohol use, were not quantified, and the type of drugs and number of drugs used for hypertension treatment were not investigated, which may impact on the measures of HRQoL to some extent. In addition, the sample size is relatively small. This study can be viewed as a case study and a generalization to other areas should be made with caution.

## 5. Conclusions

Perceived economic burden that is caused by hypertension was the most common factor that impacts on patients’ HRQoL. Female patients were more susceptible, when compared to male patients. The study suggested healthy life style, such as regular physical exercise and family members’ support, would have a positive impact on HRQoL among hypertension patients. Health intervention strategies need to be further explored and adapted to the context to improve HRQoL for patients who suffer from hypertension and other chronic non-communicable diseases.

## Figures and Tables

**Table 1:** Demographic and socioeconomic characteristics of hypertensive patients, survey in 2017.

Variable		*n*	%
Sex	Male	226	39.86
	Female	341	60.14
Age	≤59	94	16.58
	60–74	351	61.90
	≥75	122	21.52
Location	Rural	349	61.55
	Urban	218	38.45
Course of hypertension (year)	1–5	207	36.51
	>5	360	63.49
Treatment ^a^	Single treatment ^b^	261	46.03
	Various treatment methods (≥2) ^c^	306	53.97
Marital status	Married	443	78.13
	Single	12	2.12
	Divorced/widowed	112	19.75
Education ^d^	Illiteracy	206	36.33
	Primary/junior high school	325	57.32
	High school and higher	36	6.35
Employed ^e^	Yes	314	55.38
	No	253	44.62
Insurance	UEBMI	129	22.75
	URRBMI	438	77.25
Family members living together	≤2	333	58.73
	≥3	234	41.27
Income category	Low (<3600 RMB)	186	32.80
	Medium (3600–10,560 RMB)	194	34.22
	High (>10,560 RMB)	187	32.98
Perceived economic burden caused by hypertension	Low	91	16.05
	Middle	149	26.28
	High	327	57.67
Physical activity	At least once a week	413	72.84
	No	154	27.16
Smoke	Yes	92	16.23
	No	475	83.77
Alcohol	Yes	43	7.58
	No	524	92.42
Emotional self-regulation	Yes	408	71.96
	No	159	28.04

^a^ Patients were asked if they took diet control, exercise, medical treatment, and/or Chinese medicine for hypertension management and treatment; ^b^ Single treatment: one of the above four treatment methods; ^c^ Various treatment methods (≥2): two or more of the above four treatment methods; ^d^ Those who had never received or completed school education were categorized as “illiteracy”. In general, the primary school entrance age was around 6–7 years old; junior high school was around 12–13 years old and high school was around 15–16 years old; ^e^ Patients who were retired were grouped into unemployed.

**Table 2:** Factors associated with the scores of Medical Outcomes Study Short Form Health Survey questionnaire (SF-36) among participants.

Variables	Factors	β	BETA	SE	*t*	*p* *
PF	Age	7.83724	0.20561	1.49023	5.26	<0.0001
	Sex	8.83008	0.18435	1.90685	4.63	<0.0001
	Insurance	8.37670	0.14975	2.90230	2.89	0.0040
	Self-perceived economic burden caused by hypertension	7.19362	0.23036	1.21296	5.93	<0.0001
	Physical activity	9.59954	0.18207	2.06773	4.64	<0.0001
	Alcohol	−7.25962	−0.08195	3.46427	−2.10	0.0366
RP	Location	11.65367	0.12308	3.82828	3.04	0.0024
	Education	14.39468	0.18140	3.15188	4.57	<0.0001
	Self-perceived economic burden caused by hypertension	14.10584	0.22999	2.38992	5.90	<0.0001
	Physical activity	9.84206	0.09504	4.07352	2.42	0.0160
	Alcohol	−23.01350	−0.13228	6.91768	−3.33	0.0009
BP	Age	2.79476	0.09181	1.22662	2.28	0.0231
	Sex	4.47809	0.11707	1.57062	2.85	0.0045
	Insurance	5.30729	0.11881	2.38457	2.23	0.0264
	Self-perceived economic burden caused by hypertension	5.07383	0.20346	0.99828	5.08	<0.0001
	Alcohol	−5.61383	−0.07936	2.85002	−1.97	0.0494
GH	Course of hypertension	3.26230	0.08918	1.44498	2.26	0.0243
	Education	3.35821	0.11067	1.22914	2.73	0.0065
	Insurance	3.57809	0.08517	1.73104	2.07	0.0392
	Self-perceived economic burden caused by hypertension	5.62434	0.23981	0.94052	5.98	<0.0001
	Alcohol	−5.75528	−0.08651	2.63412	−2.18	0.0293
	Emotional self-regulation	4.36437	0.11131	1.56993	2.78	0.0056
VT	Sex	3.62593	0.09244	1.62195	2.24	0.0258
	Insurance	5.65417	0.12343	2.07809	2.72	0.0067
	Income category	2.12625	0.08980	1.07526	1.98	0.0485
	Self-perceived economic burden caused by hypertension	6.36889	0.24905	1.02317	6.22	<0.0001
SF	Sex	5.09871	0.11311	1.91781	2.66	0.0081
	Economic burden of hypertension	4.63086	0.15757	1.20565	3.84	0.0001
	Alcohol	−7.76904	−0.09319	3.48916	−2.23	0.0264
RE	Location	14.80345	0.15947	3.77104	3.93	<0.0001
	Self-perceived economic burden caused by hypertension	10.67508	0.17753	2.44563	4.36	<0.0001
	Alcohol	−16.50909	−0.09678	7.04800	−2.34	0.0195
MH	Sex	4.23116	0.11362	1.48788	2.84	0.0046
	Treatment	3.01691	0.08247	1.48899	2.03	0.0432
	Self-perceived economic burden caused by hypertension	5.87608	0.24202	0.98918	5.94	<0.0001
	Emotional self-regulation	3.64788	0.08987	1.63416	2.23	0.0260
PCS	Age	3.53404	0.10699	1.29707	2.72	0.0066
	Sex	4.29669	0.10352	1.68420	2.55	0.0110
	Education	4.71062	0.13455	1.44752	3.25	0.0012
	Insurance	7.55796	0.15592	1.86144	4.06	<0.0001
	Self-perceived economic burden caused by hypertension	7.92674	0.29293	1.01058	7.84	<0.0001
	Physical activity	6.04096	0.13222	1.69411	3.57	0.0004
	Alcohol	−10.62399	−0.13840	2.88293	−3.69	0.0003
MCS	Insurance	6.11371	0.12758	1.95083	3.13	0.0018
	Self-perceived economic burden caused by hypertension	6.98583	0.26114	1.06693	6.55	<0.0001
	Alcohol	−7.12307	−0.09387	3.04094	−2.34	0.0195

* Significant *p* (*p* < 0.05).

**Table 3:** Factors associated with the SF-36 scores stratified by sex, multiple liner regression.

Variables		Men		Women	
		β	*p* *	β	*p* *
PF	Age	−	−	8.00824	<0.0001
	Family members living together	−	−	4.91059	0.0445
	Self-perceived economic burden caused by hypertension	9.20862	<0.0001	6.79047	<0.0001
	Physical activity	8.27001	0.0088	9.83381	0.0003
RP	Location	−	−	19.38252	<0.0001
	Education	−	−	5.64705	0.0387
	Insurance	14.06069	0.0455	−	−
	Family members living together	−	−	13.29323	0.0040
	Self-perceived economic burden caused by hypertension	12.30444	0.0026	15.43663	<0.0001
	Physical activity	15.91683	0.0167	−	−
	Alcohol	−23.11539	0.0063	−	−
BP	Age	−	−	3.97340	0.0092
	Location	8.03367	0.0021	−	−
	Insurance	−	−	7.85190	0.0008
	Self-perceived economic burden caused by hypertension	5.64928	0.0006	4.72231	0.0002
	Physical activity	7.25903	0.0090	−	−
GH	Education	−	−	2.72569	0.0083
	Insurance	7.53731	0.0068	−	−
	Self-perceived economic burden caused by hypertension	5.19251	0.0011	5.81299	<0.0001
	Alcohol	−7.21157	0.0270	−	−
	Emotional self-regulation	5.57995	0.0313	−	−
VT	Insurance	8.47617	0.0186	−	−
	Income category	4.15787	0.0256	−	−
	Self-perceived economic burden caused by hypertension	6.23001	0.0001	6.50171	<0.0001
	Physical activity	−	−	4.22616	0.0592
SF	Self-perceived economic burden caused by hypertension	4.84363	0.0132	4.10999	0.0081
	Physical activity	8.87981	0.0071	−	−
	Alcohol	−8.40125	0.0415	−	−
RE	Location	−	−	20.07046	<0.0001
	Family members living together	−	−	12.20182	0.0117
	Self-perceived economic burden caused by hypertension	10.74271	0.0048	11.85178	0.0002
	Physical activity	14.52730	0.0218	−	−
	Alcohol	−18.67523	0.0211	−	−
MH	Income category	9.16073	0.0004	−	−
	Treatment	−	−	4.96459	0.0120
	Self-perceived economic burden caused by hypertension	6.70308	<0.0001	5.62970	<0.0001
	Emotional self-regulation	−	−	3.84501	0.0734
PCS	Age	−	−	4.16804	0.0147
	Insurance	−	−	7.80464	0.0019
	Family members living together	−	−	3.98402	0.0433
	Income category	8.53442	0.0039	−	−
	Self-perceived economic burden caused by hypertension	8.02238	<0.0001	8.04824	<0.0001
	Physical activity	8.25833	0.0031	4.89480	0.0255
	Alcohol	−10.94985	0.0020	−	−
MCS	Location	−	−	6.39821	0.0027
	Income category	6.67924	0.0219	−	−
	Self-perceived economic burden caused by hypertension	7.24929	<0.0001	7.14114	<0.0001
	Physical activity	6.91718	0.0120	−	−
	Alcohol	−7.97098	0.0225	−	−

* Significant *p* (*p* < 0.05).
